# Supplementary material for: Conserved role of hnRNPL in alternative splicing of epigenetic modifiers enables B cell activation
Source: EMBO Rep. 2024 May 14;25(6):12. doi: 10.1038/s44319-024-00152-3 (PMC11169469; doi:10.1038/s44319-024-00152-3)
Supplement: Supplementary file 7 — Expanded View Figures [file 44319_2024_152_MOESM7_ESM.pdf]

Expanded View Figures

Figure EV1. Characterization of mice with *Hnrnpl* deletion in B cells (Related to Figs. 1 and 2).

(**A**) Body and spleen weights in *CD21-cre* (+/+), *CD21-cre Hnrnpl*$^{F/+}$ (F/+) and *CD21-cre Hnrnpl*$^{F/F}$ (F/F) mice 11 days post-immunization with NP-OVA. Data compiled from multiple mice (n = as indicated) and 4 experiments. (**B**) Splenic B and T cell counts for the mice in (**A**). (**C**) GC B cell counts for the mice in (**A**). (**D**) Gating and representative flow cytometry plot and proportions of splenic NF, MZ, FO, and GC B cell subpopulations for the mice in (**A**). (**E**) Body and spleen weights and splenocyte counts, of irradiated mice that received BM cells from µ*MT* mice and either *CD21-cre Hnrnpl*$^{F/+}$ (F/+) or *CD21-cre Hnrnpl*$^{F/F}$ (F/F) mice, (n = 9 F/+, n = 8 F/F. Data compiled from two experiments. (**F**) Initial proportion of CD45.1:CD45.2 BM cell mixes from CD45.1 WT and CD45.2 either *Rosa*$^{mT/mG}$ *CD21-cre Hnrnpl*$^{F/+}$ (F/+) or *Rosa*$^{mT/mG}$ *CD21-cre Hnrnpl*$^{F/F}$ (F/F), used for reconstituting irradiated C57BL6/J CD45.2 mice. (**G**) Total B cell counts (symbols are individual mice) and proportion of splenic B cell subpopulations (with representative flow cytometry plots below) in mice reconstituted with BM mixes from (F), n = 8 mice per group, from two experiments. (**H**) Representative flow cytometry plots of the proportions of splenic B cell subpopulations newly formed (NF), marginal zone B (MZ), follicular B (Fo), and germinal center (GC) B cells in each group of mice reconstituted with BM mixes from (**F**). Data information: In (**A**–**E**, **G** leftmost panel), data were presented for individual mice with lines indicating mean values. In (**G**), data were presented as mean ± SD. Statistical tests were: (**A**–**D**) one-way ANOVA with post hoc Tukey's multiple comparison test, (**E**) unpaired two-tailed *t*-test with Welch's correction, (**G**) unpaired, two-tailed Mann–Whitney test, with $p < 0.05$ considered as significant differences in group means.

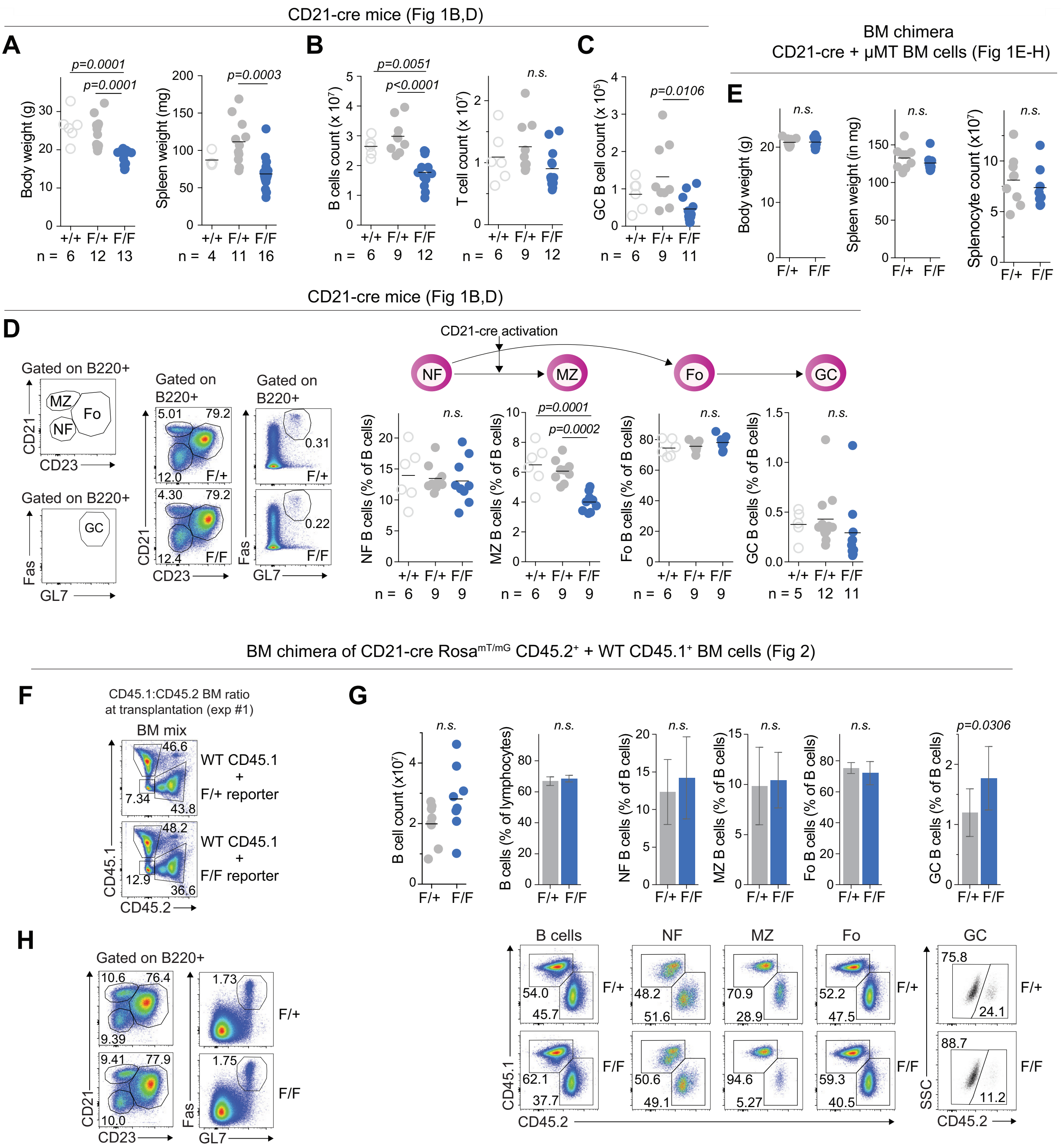
CD21-cre mice (Fig 1B,D)
A
B
C
BM chimera
CD21-cre + μMT BM cells (Fig 1E-H)
E
p=0.0001
p=0.0001
Body weight (g)
p=0.0003
Spleen weight (mg)
p=0.0051
p<0.0001
B cells count (x 10^7)
n.s.
T cell count (x 10^7)
p=0.0106
GC B cell count (x 10^5)
+/+ F/+ F/F
n = 6 12 13
n = 4 11 16
n = 6 9 12
n = 6 9 12
n = 6 9 11
n.s.
Body weight (g)
Spleen weight (in mg)
Splenocyte count (x10^7)
F/+ F/F
CD21-cre mice (Fig 1B,D)
D
Gated on B220+
MZ
Fo
NF
CD21
CD23
GC
Fas
GL7
Gated on B220+
CD21-cre activation
NF
MZ
Fo
GC
NF B cells (% of B cells)
MZ B cells (% of B cells)
p=0.0001
p=0.0002
Fo B cells (% of B cells)
GC B cells (% of B cells)
n = 6 9 9
n = 5 12 11
BM chimera of CD21-cre Rosa mT/mG CD45.2+ + WT CD45.1+ BM cells (Fig 2)
F
CD45.1:CD45.2 BM ratio
at transplantation (exp #1)
BM mix
WT CD45.1
+
F/+ reporter
WT CD45.1
+
F/F reporter
CD45.1
CD45.2
G
B cell count (x10^7)
B cells (% of lymphocytes)
NF B cells (% of B cells)
MZ B cells (% of B cells)
Fo B cells (% of B cells)
GC B cells (% of B cells)
p=0.0306
B cells
NF
MZ
Fo
GC
SSC
H
Gated on B220+
CD21
CD23
Fas
GL7
F/+
F/F

 © The Author(s)

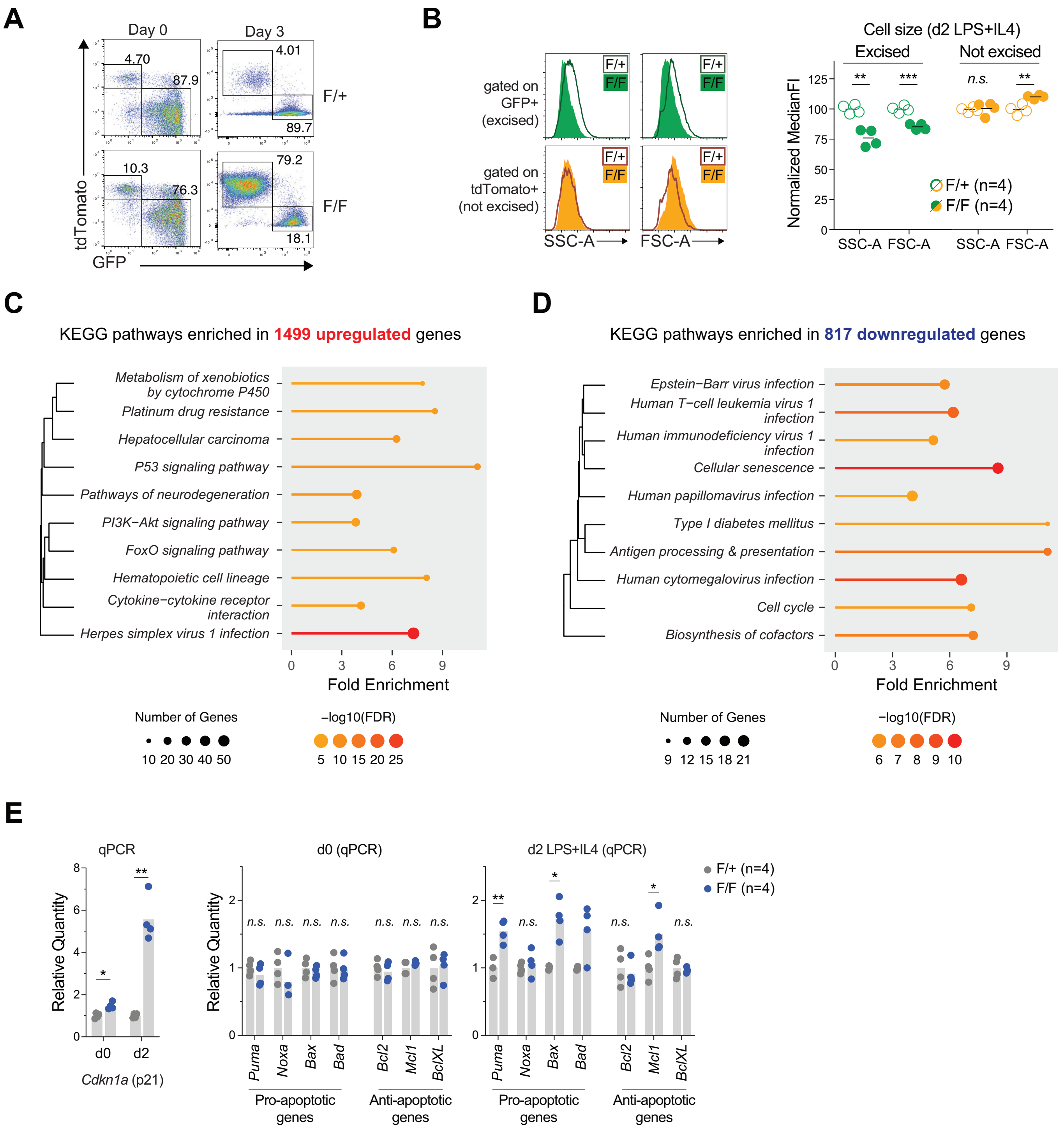

Figure EV2. Cellular and transcriptional effects of hnRNPL loss in B cells (Related to Fig. 3).

(**A**) Representative flow cytometry plots showing the proportions of hnRNPL-excised (GFP+; green) and non-excised (tdTomato$^+$; orange) splenic B cells from *Rosa*$^{mT/mG}$ *CD21-cre Hnrnpl*$^{F/+}$ (F/+) or *Rosa*$^{mT/mG}$ *CD21-cre Hnrnpl*$^{F/F}$ (F/F), before and after (day 3) ex vivo activation with LPS/IL-4. (**B**) Representative flow cytometry histograms of parameters indicating cell size (FSC) and granularity (SSC) of activated B cells from (**A**), ($n = 4$ mice per group). (**C**) Top ten KEGG pathways enriched in genes upregulated in hnRNPL-deficient versus WT cells. The dendrograms indicate the degree of similarity (shared genes) between pathways. (**D**) Top ten KEGG pathways enriched in genes downregulated in hnRNPL-deficient versus WT cells. Legends, as in (**C**). (**E**) Quantification of gene expression by RT-qPCR for p21 (*Cdkn1a*), pro- and antiapoptotic factors in B cells, resting (d0) or activated (48 h post-LPS/IL-4) (d2), from *CD21-cre Hnrnpl*$^{F/+}$ (F/+) or *CD21-cre Hnrnpl*$^{F/F}$ (F/F) mice, ($n = 4$ mice each group). Data information: In (**B**, **E**), data were presented for individual mice with lines or bars indicating mean values. Data compiled from two experiments. Statistical significance ($p < 0.05$) by unpaired, two-tailed *t*-test with Welch's correction ($*p < 0.05$; $**p < 0.01$; $***p < 0.001$).

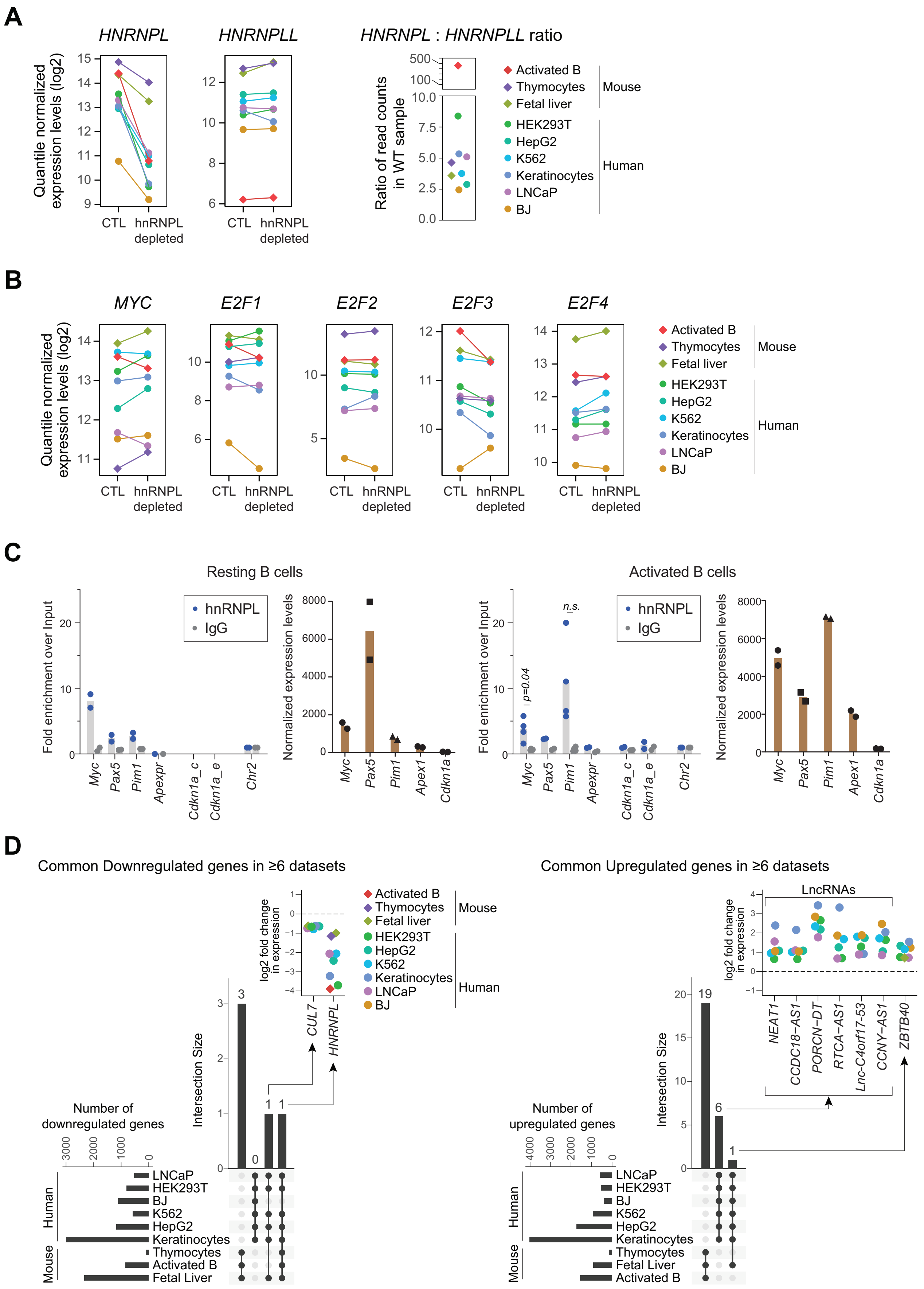
A
HNRNPL
HNRNPLL
HNRNPL : HNRNPLL ratio
Quantile normalized expression levels (log2)
Ratio of read counts in WT sample
CTL
hnRNPL depleted
Activated B
Thymocytes
Fetal liver
Mouse
HEK293T
HepG2
K562
Keratinocytes
LNCaP
BJ
Human
B
MYC
E2F1
E2F2
E2F3
E2F4
C
Resting B cells
Activated B cells
Fold enrichment over Input
Normalized expression levels
hnRNPL
IgG
n.s.
p=0.04
Myc
Pax5
Pim1
Apexpr
Cdkn1a_c
Cdkn1a_e
Chr2
Apex1
Cdkn1a
D
Common Downregulated genes in ≥6 datasets
Common Upregulated genes in ≥6 datasets
log2 fold change in expression
CUL7
HNRNPL
LncRNAs
NEAT1
CCDC18−AS1
PORCN−DT
RTCA−AS1
Lnc-C4orf17-53
CCNY−AS1
ZBTB40
Intersection Size
Number of downregulated genes
Number of upregulated genes
Fetal Liver

Figure EV3. Conserved roles of hnRNPL – (Related to Fig. 5).

(**A**) Quantile-normalized expression levels of *HNRNPL* and *HNRNPLL* in the indicated control (CTL) or hnRNPL-depleted cells, and ratio of *HNRNPL* over *HNRNPLL* read counts in each control cell type. (**B**) Quantile-normalized expression levels of the indicated genes in the various control or hnRNPL-depleted cells. (**C**) hnRNPL occupancy by ChIP-qPCR at the indicated loci in WT splenic B cells resting or activated (LPS/IL-4 for 2 days) and normalized expression levels of the corresponding genes in the same cells (GSE90094). (**D**) Comparison of significantly (adjusted p value <0.1 and fold-change ≥1.5) up- and downregulated genes upon hnRNPL depletion shared by the indicated cell types. All non-zero intersections of ≥6 datasets are shown. The insets show the relative expression ($\log_2$ fold-change compared to Control) of selected genes in the same cell types. Data information: In (**A**, **B**) data points are individual datasets, in (**C**) data points are individual mice with bars indicating means ($n = 2$ or 4 biological replicates, from two experiments). Statistical testing was done for amplicons with four replicates by two-tailed paired t-test.

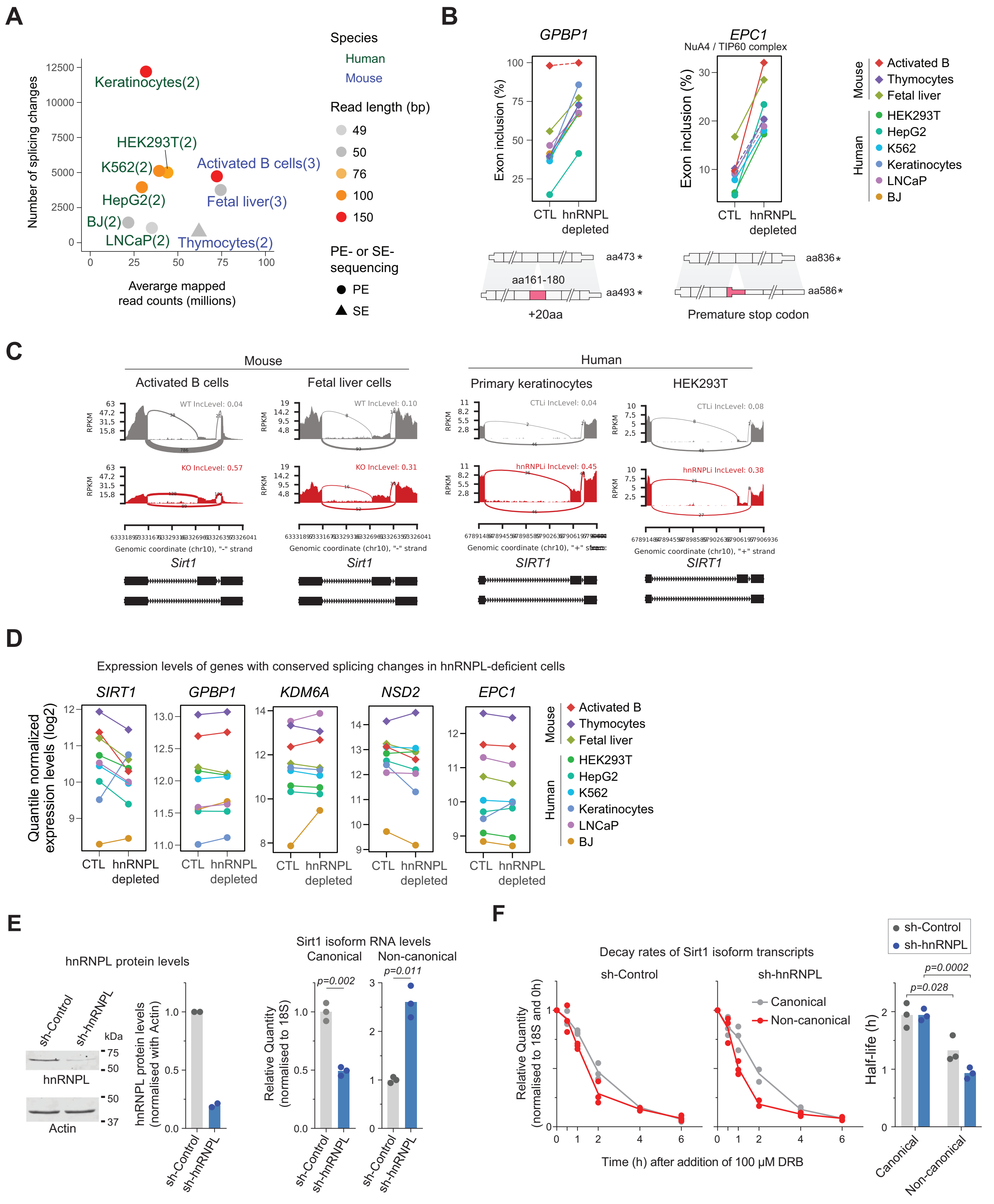
A
Number of splicing changes
Keratinocytes(2)
HEK293T(2)
K562(2)
Activated B cells(3)
HepG2(2)
Fetal liver(3)
BJ(2)
LNCaP(2)
Thymocytes(2)
Averarge mapped
read counts (millions)
Species
Human
Mouse
Read length (bp)
49
50
76
100
150
PE- or SE-
sequencing
PE
SE
B
GPBP1
EPC1
NuA4 / TIP60 complex
Exon inclusion (%)
CTL
hnRNPL
depleted
aa473 *
aa161-180
aa493 *
+20aa
aa836 *
aa586 *
Premature stop codon
Mouse
Activated B
Thymocytes
Fetal liver
Human
HEK293T
HepG2
K562
Keratinocytes
LNCaP
BJ
C
Mouse
Human
Activated B cells
Fetal liver cells
Primary keratinocytes
HEK293T
RPKM
WT IncLevel: 0.04
WT IncLevel: 0.10
CTLi IncLevel: 0.04
CTLi IncLevel: 0.08
KO IncLevel: 0.57
KO IncLevel: 0.31
hnRNPLi IncLevel: 0.45
hnRNPLi IncLevel: 0.38
Genomic coordinate (chr10), "-" strand
Genomic coordinate (chr10), "+" strand
Sirt1
SIRT1
D
Expression levels of genes with conserved splicing changes in hnRNPL-deficient cells
SIRT1
GPBP1
KDM6A
NSD2
EPC1
Quantile normalized
expression levels (log2)
CTL
hnRNPL
depleted
Mouse
Activated B
Thymocytes
Fetal liver
Human
HEK293T
HepG2
K562
Keratinocytes
LNCaP
BJ
E
hnRNPL protein levels
sh-Control
sh-hnRNPL
kDa
75
50
hnRNPL
50
37
Actin
hnRNPL protein levels
(normalised with Actin)
Sirt1 isoform RNA levels
Canonical
Non-canonical
p=0.002
p=0.011
Relative Quantity
(normalised to 18S)
F
Decay rates of Sirt1 isoform transcripts
sh-Control
sh-hnRNPL
Canonical
Non-canonical
Relative Quantity
(normalised to 18S and 0h)
Time (h) after addition of 100 µM DRB
p=0.0002
p=0.028
Half-life (h)
Canonical
Non-canonical

 © The Author(s)

Figure EV4. Comparison of hnRNPL depletion effects among cell types (Related to Fig. 6).

(**A**) Number of splicing changes detected in different hnRNPL-deficient cell types as a function of read depth. Read length and species of the datasets are indicated by codes (legends), as well as number of replicates per condition (in parenthesis). (**B**) Mean exon inclusion levels for the indicated splicing events in control (CTL) and hnRNPL-depleted cell types. The transcript schemes indicate the exons (in red, the exon more included in hnRNPL-deficient cells), positions of amino acids coded by the included exon and stop codons in the respective human transcripts. (**C**) Sashimi plot showing the inclusion of an intermediate exon in *SIRT1* regulated by hnRNPL status in selected cell types. (**D**) Quantile-normalized expression levels of indicated genes in the various CTL or hnRNPL-depleted cell types. (**E**) Representative western blot and quantification of hnRNPL protein levels in CH12-F3 cells expressing shRNA control or targeting hnRNPL ($n = 2$ biological replicates). Steady-state levels of canonical and non-canonical Sirt1 transcript isoforms measured by RT-qPCR normalized to the sh-Control ($n = 3$ biological replicates). (**F**) Decay rates and half-lives of canonical and non-canonical Sirt1 isoforms following transcription inhibition with 100 μM DRB in sh-Control and sh-hnRNPL CH12-F3 cells measured by RT-qPCR ($n = 3$ biological replicates). Data information: In (**A**, **B**, **D**), data points represent individual datasets. In (**E**, **F**), data points are biological replicates with bars indicating means. Statistical significance ($p < 0.05$) tested by unpaired two-tailed *t*-tests with Welch's correction.

© The Author(s)

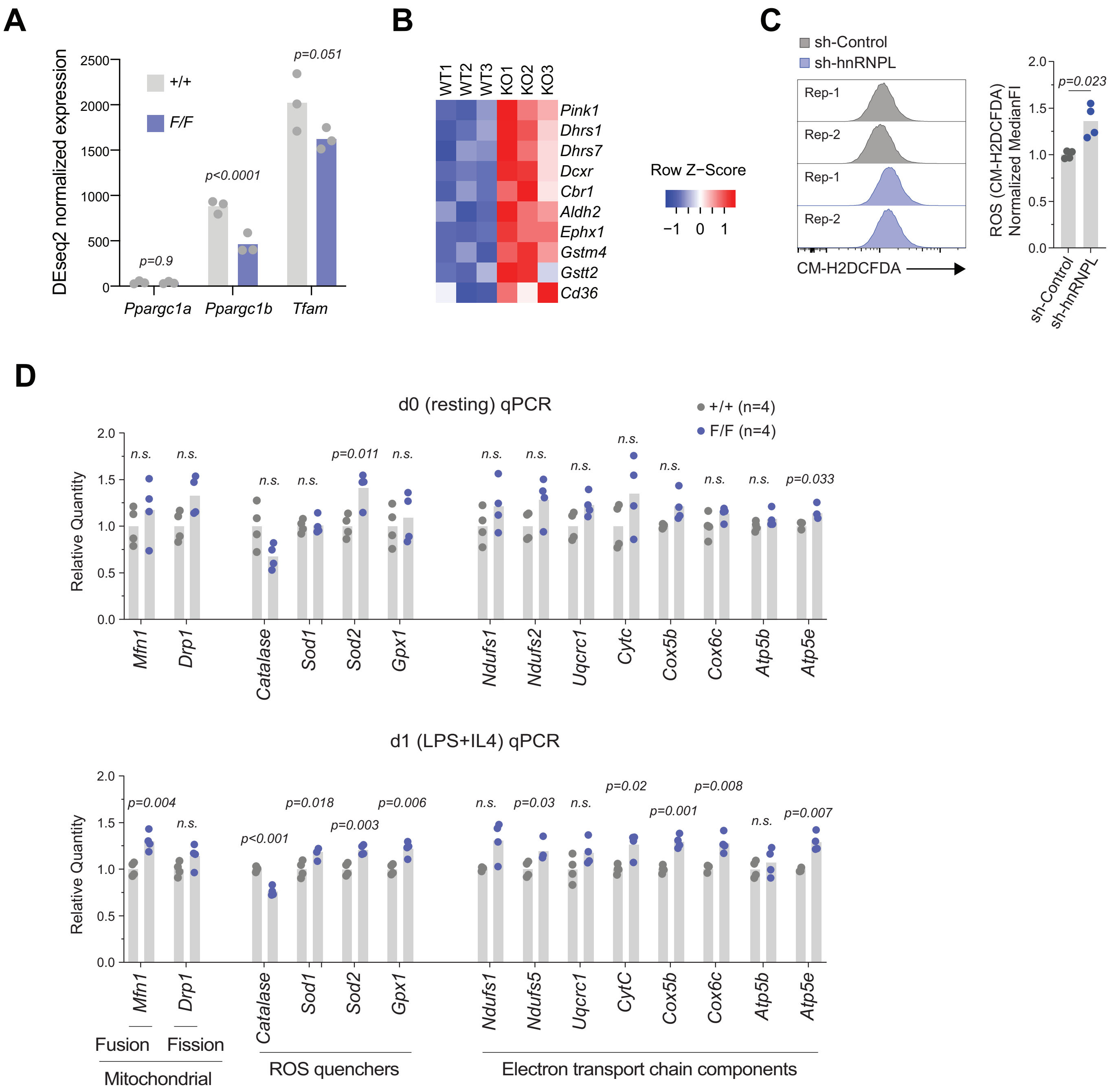

Figure EV5. Mitochondrial function-related gene expression (Related to Fig. 7).

(**A**) Expression of the indicated genes from RNA-seq of splenic B cells activated ex vivo for 1 day with LPS/IL-4 from hnRNPL-deficient (GFP+ cells from *Rosa*$^{mT/mG}$ *CD21-cre Hnrnpl*$^{F/F}$) and WT (GFP+ cells from *Rosa*$^{mT/mG}$ *CD21-cre*) mice. (**B**) Heatmap of selected genes in individual biological replicates from RNA-seq from data in Dataset EV1. (**C**) Representative flow cytometry histograms of ROS levels measured by the CM-H2DCFDA probe in CH12-F3 cells expressing shRNA targeting hnRNPL (sh-hnRNPL) or control (sh-Control). ($n = 4$ biological replicates per genotype from two experiments). (**D**) Relative expression level of selected genes measured by RT-qPCR in resting (d0) and ex vivo LPS/IL-4-activated splenic B cells from *CD21-cre* (+/+) and *CD21-cre Hnrnpl*$^{F/F}$ (F/F) mice. ($n = 4$ biological replicates per genotype from two experiments). Data information: In (**A**, **C**, **D**), data points are individual biological replicates with bars indicating means; in (**B**), row normalized data, both using values from Dataset EV1. Statistical significance (P adj <0.1 or $p < 0.05$) was tested (**A**) by Deseq2, (**C**, **D**)) by unpaired two-tailed t-tests with Welch's correction.

 © The Author(s)
